# Supplementary material for: An open-source framework for large-scale, flexible evaluation of biomedical text mining systems
Source: J Biomed Discov Collab. 2008 Jan 29;3:1. doi: 10.1186/1747-5333-3-1 (PMC2276192; doi:10.1186/1747-5333-3-1)
Supplement: Additional file 1 — Evaluation platform source code. The additional file contains the software evaluation framework discussed in this paper. The most current release can be downloaded from SourceForge.net [1]. See the accompanying README file for instructions on installation and use. Also included in the distribution is a collection of UIMA wrappers for some commonly used BioNLP tools and annotated corpora. [file 1747-5333-3-1-S1.zip › bionlp_uima_repository-1.0.1/docs/api/src-html/edu/uchsc/ccp/uima/ae/nlp/opendmap/CCPOpenDMAP_AE.html]

```
001    /**
002     * The OpenDMAP for Protege Project
003     * February 2006
004     */
005    package edu.uchsc.ccp.uima.ae.nlp.opendmap;
006    
007    import java.io.File;
008    import java.util.ArrayList;
009    import java.util.Collection;
010    import java.util.Iterator;
011    import java.util.List;
012    
013    import org.apache.uima.analysis_engine.ResultSpecification;
014    import org.apache.uima.analysis_engine.annotator.AnnotatorContext;
015    import org.apache.uima.analysis_engine.annotator.AnnotatorContextException;
016    import org.apache.uima.analysis_engine.annotator.AnnotatorInitializationException;
017    import org.apache.uima.jcas.JCas;
018    import org.apache.uima.jcas.cas.FSArray;
019    import org.apache.uima.jcas.cas.StringArray;
020    
021    import edu.uchsc.ccp.opendmap.DMAPItem;
022    import edu.uchsc.ccp.opendmap.DMAPToken;
023    import edu.uchsc.ccp.opendmap.InfoPacket;
024    import edu.uchsc.ccp.opendmap.Parser;
025    import edu.uchsc.ccp.opendmap.ProtegeFrameItem;
026    import edu.uchsc.ccp.opendmap.Reference;
027    import edu.uchsc.ccp.uima.annotation.CCPAnnotator;
028    import edu.uchsc.ccp.uima.annotation.CCPDocumentInformation;
029    import edu.uchsc.ccp.uima.annotation.CCPSpan;
030    import edu.uchsc.ccp.uima.annotation.CCPTextAnnotation;
031    import edu.uchsc.ccp.uima.mention.CCPClassMention;
032    import edu.uchsc.ccp.uima.mention.CCPComplexSlotMention;
033    import edu.uchsc.ccp.uima.mention.CCPNonComplexSlotMention;
034    import edu.uchsc.ccp.uima.mention.CCPSlotMention;
035    import edu.uchsc.ccp.uima.util.opendmap.AnnotationMap;
036    import edu.uchsc.ccp.uima.util.opendmap.DMAPJcasTokenizer;
037    import edu.uchsc.ccp.uima.util.opendmap.DMAPStanfordPhraseGraph;
038    import edu.uchsc.ccp.uima.util.opendmap.ReferenceProcessor;
039    
040    /**
041    * This class packages OpenDMAP into the UIMA framework.
042     * <p>
043     * This annotator assumes that the input GeneRIF can be found in
044     * the JCas in CCPTextAnnotation structures and copies the output
045     * references of interest into the JCas as CCPTextAnntotion
046     * structures.
047     * 
048     * @author R. James Firby
049     */
050    public class CCPOpenDMAP_AE extends OpenDmapAnnotator {
051    
052            /* These are the parameter names exposed in the UIMA dscriptor for this annotator */
053            private static final String PARAM_IGNOREPHRASECONSTAINTS = "IgnorePhraseConstraints";
054            private static final String PARAM_DEBUG = "PrintDebugInformation";
055            private static final String PARAM_CONTEXTFILE = "PipelineContextFile";
056            
057            /* The default setting for the 'Print Debug Information' parameter */
058            private boolean debug = false;
059            /* The deault value for the 'Ignore Phrase Constraints' parameter */
060            private boolean ignorePhraseConstraints = false;
061            
062            /* The annotation map that maps from input JCas annotations to DMAP tokens and
063             * from DMAP output references back to JCas annotations.
064             */
065            private AnnotationMap annotationMap = null;
066            
067            /**
068             * Create a new GeneRIF Annotator that uses OpenDMAP to
069             * process the input document text.
070             */
071            public CCPOpenDMAP_AE() {
072                    super();
073            }
074            
075            /**
076             * Initialize the GeneRIF Annotator.  Get the parameter values, load
077             * the annotation map, and create the parser.
078             */
079            public void initialize(AnnotatorContext context) throws AnnotatorInitializationException {
080                    // See if we should ignore phrase constraints
081                    ignorePhraseConstraints = false;
082            Boolean param = null;
083                    try {
084                            param = (Boolean) context.getConfigParameterValue(PARAM_IGNOREPHRASECONSTAINTS);
085                    } catch (AnnotatorContextException e) {}
086            if (param != null)
087                    ignorePhraseConstraints = param.booleanValue();
088            
089            // See if we should print debugging information
090                    debug = false;
091            param = null;
092                    try {
093                            param = (Boolean) context.getConfigParameterValue(PARAM_DEBUG);
094                    } catch (AnnotatorContextException e) {}
095            if (param != null)
096                    debug = param.booleanValue();
097            
098            // Setup input and output mapping information
099            String contextFile = null;
100            try {
101                    contextFile = (String) context.getConfigParameterValue(PARAM_CONTEXTFILE);
102            } catch (AnnotatorContextException e) {}
103            if (contextFile == null) {
104                    annotationMap = new AnnotationMap();
105            } else {
106                    try {
107                                    annotationMap = new AnnotationMap(contextFile);
108                            } catch (Exception e) {
109                                    throw new AnnotatorInitializationException("Cannot load GenerifAnnotator context file '" + new File(contextFile) + "'", new Object[]{this}, (Throwable) e);
110                            }
111            }
112            
113            // Initialize the parser
114            super.initialize(context);
115            }
116    
117            /**
118             * Process the JCas using the annotation map to help extract input tokens.
119             * Write the references found back into the JCas using the annotation map to
120             * describe the desired output annotations.
121             */
122            @Override
123            public void process(Parser parser, JCas jcas, ResultSpecification resultSpec) {
124                    // A debugging heading
125                    if (debug) {
126                Iterator docInfoIter = jcas.getJFSIndexRepository().getAnnotationIndex(CCPDocumentInformation.type).iterator();
127                String docID = "-1";
128                if (docInfoIter.hasNext()) {
129                    CCPDocumentInformation docInfo = (CCPDocumentInformation) docInfoIter.next();
130                    docID = docInfo.getDocumentID();
131                }
132                            System.out.println("---------------------------------------------------------------------------");
133                            System.out.println(docID + ": " + jcas.getDocumentText());
134                            System.out.println();
135                            System.out.println("Annotation Context Map");
136                            if (annotationMap != null) annotationMap.print(System.out, 2);
137                    }
138                    // Get the input tokens from the JCas
139                    DMAPJcasTokenizer tokenizer = new DMAPJcasTokenizer(parser, jcas, annotationMap, debug);
140                    // Get the Stanford Parser Phrase Annotations as a graph
141                    DMAPStanfordPhraseGraph tree = null;
142                    if (!ignorePhraseConstraints) {
143                            tree = new DMAPStanfordPhraseGraph(jcas, debug);
144                    }
145                    if (debug) {
146                            if (tree != null) {
147                                    System.out.println("\nStanford Parser Phrase Graph:");
148                                    tree.print(System.out);
149                            }
150                            System.out.flush();
151                    }
152                    // Reset the parser
153                    parser.reset();
154                    // Parse the utterance
155                    parser.parse(tokenizer, tree);
156                    // Extract the best matches
157                    Collection<Reference>     references = ReferenceProcessor.getRecognizedReferences(parser, annotationMap.getReferencesOfInterest(), debug);
158                    // Write them back to the JCas
159                    ArrayList<Reference> seen = new ArrayList<Reference>();
160                    for (Reference ref: references) {
161                            // Keep only the first (highest scoring) reference for each root
162                            if (!isSeenReference(seen, ref, annotationMap)) {
163                                    // Create the skeleton annotation
164                                    CCPTextAnnotation annotation = annotationFromReference(ref, jcas, annotationMap);
165                        // Generate the class mention associated with this annotation
166                        CCPClassMention mention = mentionFromReference(ref, jcas, annotationMap);
167                        // Link the annotation and reference
168                        linkAnnotationToMention(annotation, mention, jcas);
169                        mention.addToIndexes();
170                        annotation.addToIndexes();
171                        // This becomes a known root
172                        seen.add(ref);
173                            }
174                    }
175            }
176            
177            /**
178             * Create the cross-links needed between a CCPTextAnnotation and the CCPClassMention
179             * that was recognized for that annotation.
180             * 
181             * @param annotation The text annotation..
182             * @param mention The mention recognized using that annotation.
183             * @param jcas The JCas holding the annotation and mention.
184             */
185            private void linkAnnotationToMention(CCPTextAnnotation annotation, CCPClassMention mention, JCas jcas) {
186                    FSArray oldAnnotations = mention.getCcpTextAnnotations();
187                    FSArray newAnnotations = null;
188                    if (oldAnnotations == null) {
189                            newAnnotations = new FSArray(jcas, 1);
190                            newAnnotations.set(0, annotation);
191                    } else {
192                            newAnnotations = new FSArray(jcas, oldAnnotations.size()+1);
193                            for (int i=0; i<oldAnnotations.size(); i++) newAnnotations.set(i, oldAnnotations.get(i));
194                            newAnnotations.set(oldAnnotations.size(), annotation);
195                    }
196                    mention.setCcpTextAnnotations(newAnnotations);
197                    annotation.setClassMention(mention);
198            }
199            
200            /**
201             * Get the list of text spans from the original text that were used to recognize a reference.
202             * 
203             * @param r The reference from OpenDMAP.
204             * @param annotationMap The annotation map describing the output annotations of interest.
205             * @param jcas The JCas holding the original input text and annotations.
206             * @return A set of text spans including the text and the start and end character positions for each.
207             */
208            private ArrayList<CCPSpan> getSpanList(Reference r, AnnotationMap annotationMap, JCas jcas) {
209                    ArrayList<CCPSpan> spans = new ArrayList<CCPSpan>();
210                    // Get the root reference
211                    Reference root = annotationMap.getOutputReferenceRoot(r);
212                    //if (r.isa("c-bioprocess") && r.hasSlotValue("action")) {
213                    if (r != root) {
214                            // Treat this sort of reference special
215                            //Reference action = r.getSlotValue("action");
216                            addTokenSpans(spans, root, null, jcas);
217                    } else {
218                            // A generic span generator
219                            addTokenSpans(spans, root, annotationMap, jcas);
220                    }
221                    // Return the spans
222                    return spans;
223            }
224            
225            /**
226             * Combine input tokens in continuous spans for use in constructing the output annotations.
227             * 
228             * @param spans The span list being added to.
229             * @param reference The reference that was recognized.
230             * @param annotationMap The annotation map identifying output classes of interest.
231             * @param jcas The JCas holding the input and output annotations.
232             */
233            private void addTokenSpans(ArrayList<CCPSpan> spans, Reference reference, AnnotationMap annotationMap, JCas jcas) {
234                    CCPSpan current = null;
235                    int next = 0;
236                    for (DMAPToken token: reference.getTokens()) {
237                            if ((annotationMap == null) || !tokenUsedInSlot(token, reference, annotationMap)) {
238                                    if (current == null) {
239                                            // The very first span
240                                            current = new CCPSpan(jcas);
241                                            current.setSpanStart(token.getCharacterStart());
242                                            current.setSpanEnd(token.getCharacterEnd());
243                                            next = token.getEnd() + 1;
244                                    } else if (token.getStart() == next) {
245                                            // Extend this span
246                                            current.setSpanEnd(token.getCharacterEnd());
247                                            next = token.getEnd() + 1;
248                                    } else {
249                                            // Need to start a new span
250                                            spans.add(current);
251                                            current = new CCPSpan(jcas);
252                                            current.setSpanStart(token.getCharacterStart());
253                                            current.setSpanEnd(token.getCharacterEnd());
254                                            next = token.getEnd() + 1;
255                                    }
256                            }
257                    }
258                    if (current != null) spans.add(current);        
259            }
260            
261            /**
262             * Check whether a token was used to recognize a slot filler or just the enclosing reference.
263             * 
264             * @param token The token to check.
265             * @param r The enclosing reference.
266             * @param annotationMap The annotation map giving the slots of interest.
267             * @return True if the token was used to recognize a slot filler of interest.
268             */
269            private boolean tokenUsedInSlot(DMAPToken token, Reference r, AnnotationMap annotationMap) {
270                    if (r.getInformation() == null) return false;
271                    for (InfoPacket pair: r.getInformation()) {
272                            Reference value = pair.getValue();
273                            if (annotationMap.isOutputReferenceOfInterest(value)) {
274                                    for (DMAPToken tok: value.getTokens()) {
275                                            if (tok == token) return true;
276                                    }
277                            } else {
278                                    if (tokenUsedInSlot(token, value, annotationMap)) return true;
279                            }
280                    }
281                    return false;
282            }
283            
284            /**
285             * Check whether this type of reference has already been put into the JCas.  Only the
286             * instance of a reference with the highest score is output.
287             * 
288             * @param seen A list of references that have already been output.
289             * @param ref The reference to check.
290             * @param annotationMap The annotation map holding output classes of interest.
291             * @return True if the reference being checked has already been output.
292             */
293            private boolean isSeenReference(ArrayList<Reference> seen, Reference ref, AnnotationMap annotationMap) {
294                    Reference root = annotationMap.getOutputReferenceRoot(ref);
295                    for (Reference old: seen) {
296                            //System.out.println("Root: " + root + " - " + ref);
297                            if (annotationMap.getOutputReferenceRoot(old) == root) {
298                                    if (ReferenceProcessor.calculateScore(old) > ReferenceProcessor.calculateScore(ref)) {
299                                            return true;
300                                    }
301                            }
302                    }
303                    return false;
304            }
305            
306            /**
307             * Generate a JCas CCPTextAnnotation from an OpenDMAP output reference.
308             * 
309             * @param r The reference to write to the JCas.
310             * @param jcas The JCas to hold the annotation for this reference.
311             * @param annotationMap The annotation map describing slot mappings.
312             * @return The new CCPTextAnnotation corresponding to the reference.
313             */
314            private CCPTextAnnotation annotationFromReference(Reference r, JCas jcas, AnnotationMap annotationMap) {
315                    CCPTextAnnotation annotation = new CCPTextAnnotation(jcas);
316                    // Generate the annotator information for this annotation
317                    CCPAnnotator annotator = new CCPAnnotator(jcas);
318                    annotator.setAnnotatorID(15); // ?
319                    annotator.setFirstName("OpenDMAP");
320                    annotator.setLastName("GenerifAnnotator");
321                    annotator.addToIndexes();
322                    annotation.setAnnotator(annotator);
323                    // Generate all the span information
324                    List<CCPSpan> spans = getSpanList(r, annotationMap, jcas);
325                    if (spans.isEmpty()) {
326                            CCPSpan span = new CCPSpan(jcas);
327                            span.setSpanStart(r.getCharacterStart());
328                            span.setSpanEnd(r.getCharacterEnd());
329                            spans.add(span);
330                    }
331                    FSArray casSpans = new FSArray(jcas, spans.size());
332                    for (int i=0; i<spans.size(); i++) {
333                            casSpans.set(i, spans.get(i));
334                    }
335                    annotation.setSpans(casSpans);
336                    annotation.setBegin(spans.get(0).getSpanStart());
337                    annotation.setEnd(spans.get(spans.size()-1).getSpanEnd());
338                    // Return the skeleton annotation
339                    return annotation;
340            }
341            
342            /**
343             * Create a CCPClassMention for an OpenDMAP output reference.
344             * 
345             * @param r The output reference.
346             * @param jcas The JCas to hold the new class mention.
347             * @param annotationMap The annotation map holding slot name translations.
348             * @return The new CCPClassMention for the reference.
349             */
350            private CCPClassMention mentionFromReference(Reference r, JCas jcas, AnnotationMap annotationMap) {
351                    // See if we have a cached mention that covers us already
352                    List<DMAPToken> tokens = r.getTokens();
353                    if (tokens != null) {
354                            // Look for a single cached annotation
355                            CCPTextAnnotation cachedAnnotation = null;
356                            List<InfoPacket> cachedSlots = null;
357                            for (DMAPToken token: tokens) {
358                                    Object source = token.getSource();
359                                    if ((source != null) && (source instanceof CCPTextAnnotation)) {
360                                            if (cachedAnnotation == null)   {
361                                                    // No cached one yet
362                                                    cachedAnnotation = (CCPTextAnnotation) source;
363                                                    cachedSlots = token.getInformation();
364                                            } else {
365                                                    // Multiple cached annoations, we need to build a new one
366                                                    cachedAnnotation = null;
367                                                    cachedSlots = null;
368                                                    break;
369                                            }
370                                    }
371                            }
372                            if (cachedAnnotation != null) {
373                                    // Okay, this reference arose from a single external CCPTextAnnotation
374                                    // See if we have added any information to it
375                                    List<InfoPacket> pairs = r.getInformation();
376                                    if ((pairs == null) && (cachedSlots == null)) {
377                                            // No new information
378                                            return cachedAnnotation.getClassMention();
379                                    } else if ((pairs != null) && (cachedSlots != null) && (pairs.size() == cachedSlots.size())) {
380                                            boolean match = true;
381                                            for (InfoPacket a: pairs) {
382                                                    boolean found = false;
383                                                    for (InfoPacket b: cachedSlots) {
384                                                            if (a.getKey().equals(b.getKey())) { found = true; break; }
385                                                    }
386                                                    if (!found)     { match = false; break; }
387                                            }
388                                            if (match) {
389                                                    for (InfoPacket a: cachedSlots) {
390                                                            boolean found = false;
391                                                            for (InfoPacket b: pairs) {
392                                                                    if (a.getKey().equals(b.getKey())) { found = true; break; }
393                                                            }
394                                                            if (!found)     { match = false; break; }
395                                                    }
396                                            }
397                                            // No new information, return the cached mention
398                                            if (match) return cachedAnnotation.getClassMention();
399                                    }
400                            }
401                    }
402                    // Okay, not a cached mention, create a new one
403                    CCPClassMention mention = new CCPClassMention(jcas);
404                    mention.setMentionName(annotationMap.getOutputReferenceMentionType(r));
405        List<InfoPacket> slotInfo = r.getInformation();
406        if ((slotInfo != null) && !slotInfo.isEmpty()) {
407                ArrayList<CCPSlotMention> slots = new ArrayList<CCPSlotMention>();
408                for (int i=0; i<slotInfo.size(); i++) {
409                    InfoPacket pair = slotInfo.get(i);
410                    if (annotationMap.isOutputReferenceSlotOfInterest(r, pair.getKey())) {
411                            DMAPItem value = pair.getValue().getItem();
412                            if (value instanceof ProtegeFrameItem) {
413                                    CCPComplexSlotMention slot = new CCPComplexSlotMention(jcas);
414                                    slot.setMentionName(annotationMap.getOutputReferenceSlotMentionType(r, pair.getKey()));
415                                    CCPClassMention slotFiller = mentionFromReference(pair.getValue(), jcas, annotationMap);
416                                    CCPTextAnnotation annotation = annotationFromReference(pair.getValue(), jcas, annotationMap);
417                                    linkAnnotationToMention(annotation, slotFiller, jcas);
418                                    FSArray values = new FSArray(jcas, 1);
419                                    values.set(0, slotFiller);
420                                    slot.setClassMentions(values);
421                                    slots.add(slot);
422                            } else {
423                                    CCPNonComplexSlotMention slot = new CCPNonComplexSlotMention(jcas);
424                                    slot.setMentionName(annotationMap.getOutputReferenceSlotMentionType(r, pair.getKey()));
425                                    StringArray values = new StringArray(jcas, 1);
426                                    values.set(0, value.getText());
427                                    slot.setSlotValues(values);
428                                    slots.add(slot);
429                            }
430                    }
431                }
432                if (!slots.isEmpty()) {
433                    FSArray jcasSlots = new FSArray(jcas, slots.size());
434                    for (int i=0; i<slots.size(); i++) jcasSlots.set(i, slots.get(i));
435                    mention.setSlotMentions(jcasSlots);
436                }
437        }
438        // TODO If all is well, there should have been an annotation built somewhere
439        //  for this mention.  We should look it up somehow and stick it into the mention.
440        //CCPTextAnnotation annotation = annotationFromReference(r, jcas, interests);
441        //linkAnnotationToMention(annotation, mention, jcas);
442        //annotation.addToIndexes();
443        return mention;
444            }
445            
446    }
447    
448
```
